# Intensity and mechanisms of deltamethrin and permethrin resistance in *Anopheles gambiae* s.l. populations in southern Benin

**DOI:** 10.1186/s13071-021-04699-1

**Published:** 2021-04-14

**Authors:** Hermann Watson Sagbohan, Casimir D. Kpanou, Razaki Osse, Fortuné Dagnon, Germain G. Padonou, André Aimé Sominahouin, Albert Sourou Salako, Aboubakar Sidick, Wilfried Sewade, Bruno Akinro, Saadani Ahmed, Daniel Impoinvil, Clément Agbangla, Martin Akogbeto

**Affiliations:** 1 Centre de Recherche Entomologique de Cotonou, Cotonou, Benin; 2 Faculty of Science and Technology of the University of Abomey-Calavi, Godomey, Benin; 3 National University of Agriculture of Porto-Novo, Porto-Novo, Benin; 4 Bill & Melinda Gates Foundation, Lagos, Nigeria; 5 Genetics and Biotechnology Laboratory of the UAC, Godomey, Benin; 6 US President’s Malaria Initiative, Centers for Disease Control and Prevention (CDC), Atlanta, USA; 7 US President’s Malaria Initiative, US Agency for International Development, Cotonou, Benin

**Keywords:** *An. gambiae* s.l., Insecticide resistance, Intensity, PBO, Oxidase

## Abstract

**Background:**

Insecticide resistance is threatening the effectiveness of efforts to control malaria vectors in Benin. This study explores the levels and mechanisms of insecticide resistance in *An. gambiae* s.l. to pyrethroids.

**Methods:**

Larvae were collected from August 2017 to July 2018 in five communes in southern Benin (Adjohoun, Allada, Bohicon, Cotonou, and Porto-Novo) representing diverse ecological regions, and were reared in Benin’s insectary. Two- to five-day-old female mosquitoes from each district were exposed to multiple doses of deltamethrin and permethrin (1×, 2×, 5×, and 10×) using the WHO insecticide resistance intensity bioassay. The effect of pre-exposure to the synergist, piperonyl butoxide (PBO), was also tested at different pyrethroid doses. Molecular allele frequencies of *kdr* (1014F) and *ace-1*R (119S) insecticide resistance mutations and levels of detoxification enzymes were determined for mosquitoes sampled from each study area.

**Results:**

*An. gambiae* s.l. were resistant to pyrethroid-only exposure up to 10× the diagnostic doses in all the study sites for both deltamethrin and permethrin. Mortality was significantly higher in *An. gambiae* s.l. pre-exposed to PBO followed by exposure to deltamethrin or permethrin compared to mosquitoes exposed to deltamethrin or permethrin only (*p* < 0.001). The difference in mortality between deltamethrin only and PBO plus deltamethrin was the smallest in Cotonou (16–64%) and the greatest in Bohicon (12–93%). The mortality difference between permethrin only and PBO plus permethrin was the smallest in Cotonou (44–75%) and the greatest in Bohicon (22–72%). In all the study sites, the *kdr* resistance allele (1014F) frequency was high (75–100%), while the *ace-1* resistance allele (G119S) frequency was low (0–3%). Analysis of the metabolic enzymatic activity of *An. gambiae* s.l. showed overexpression of nonspecific esterases and glutathione S-transferases (GST) in all study sites. In contrast to the PBO results, oxidase expression was low and was similar to the susceptible *An. gambiae* s.s. Kisumu strain in all sites.

**Conclusion:**

There is high-intensity resistance to pyrethroids in southern Benin. However, pre-exposure to PBO significantly increased susceptibility to the pyrethroids in the different *An. gambiae* s.l. populations sampled. The use of PBO insecticide-treated bed nets may help maintain the gains in *An. gambiae* (s.l.) control in southern Benin.

**Graphical Abstract:**

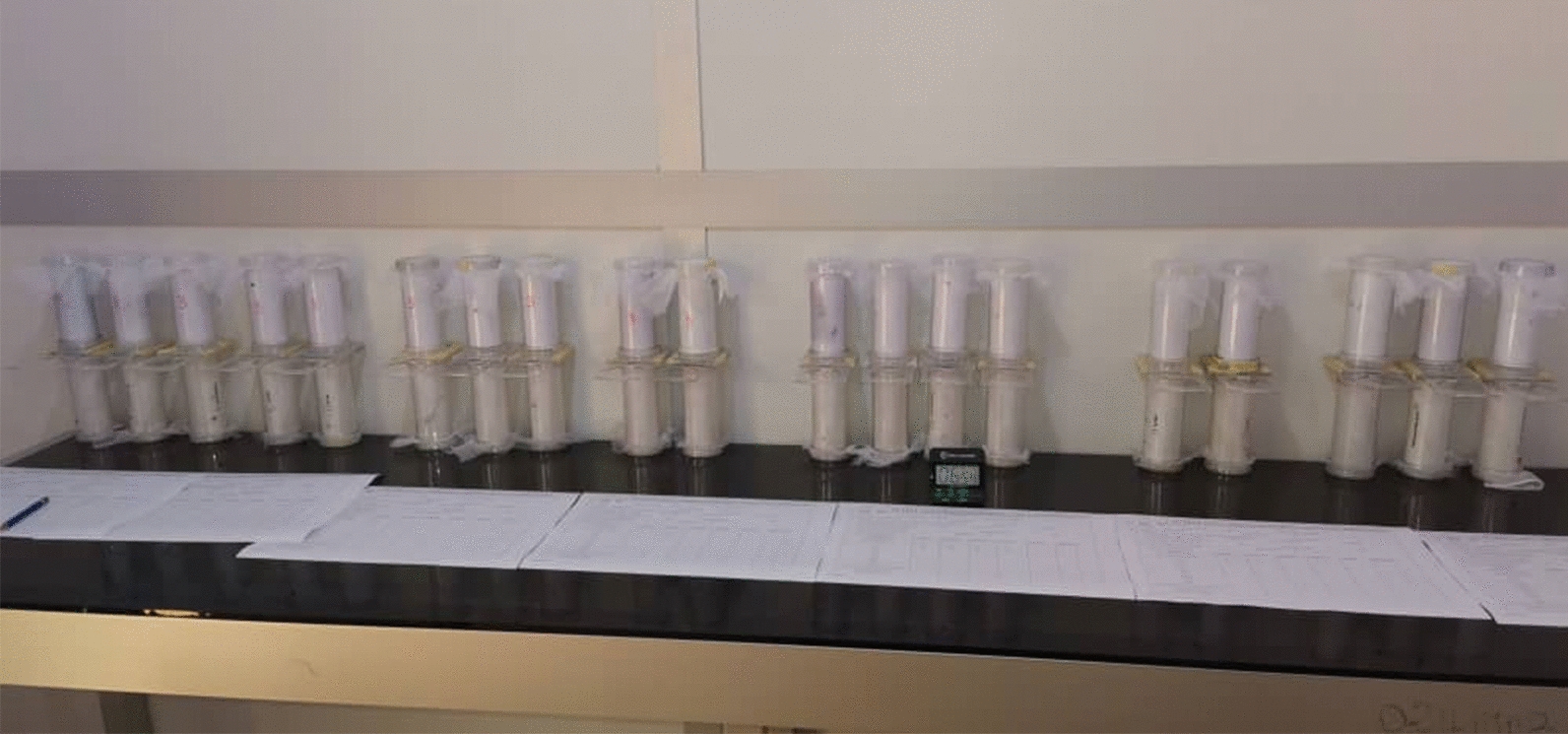

## Background

The use of insecticides through indoor residual spraying (IRS) or long-lasting insecticide-treated nets (LLINs) is a core component of vector control in all stages (i.e. control, pre-elimination, and elimination) of the global malaria elimination program [1]. In Benin, IRS and LLINs are the main vector control interventions implemented by the National Malaria Control Program (NMCP). However, while IRS is targeted in the northern part of Benin, LLINs are distributed throughout the country. Unfortunately, the effectiveness of these vector control tools, specifically LLINs, is hampered by the increasing vector resistance to pyrethroids, the principal insecticide on LLINs. Since 1999, malaria vectors in Benin have been reported to be resistant to all pyrethroids used for mosquito net impregnation, including permethrin, deltamethrin, and alpha-cypermethrin [2–10]. The most widely studied pyrethroid resistance mechanisms of *An. gambiae* s.l. are (1) modification of the insecticide target site by *kdr* mutation [11–13] and (2) overexpression of the detoxification enzymes using oxidases or glutathione S-transferases (GSTs) [14–16]. The *kdr* insecticide resistance mutation—a replacement of leucine by phenylalanine in the *kdr* 1014 gene (L1014F)—is widespread in West Africa [17–19], while the replacement with serine (L1014S) is widespread in some regions of Central and East Africa [20–22].

While insecticide resistance monitoring at the national level has shown an increase in phenotypic resistance and higher frequencies of resistance alleles in malaria vectors in some parts of the country [7, 9, 23], there are limited published reports on the intensity of resistance to insecticides used for vector control in Benin’s vector population (*An. gambiae* s.l. and *An. funestus* s.l.). To mitigate the negative impacts of higher levels of insecticide resistance, a new generation of mosquito insecticide-treated nets (ITNs) has been developed with a combination of pyrethroids and a synergist, piperonyl butoxide (PBO). Some studies have found PBO effective in increasing mortality in resistant mosquitoes in southern Benin [14, 24, 25]. However, additional studies in different locations or in different years are still useful to better characterize the spatial and temporal landscape of resistance occurrence.

There has been a global rise in (1) the frequency of resistance alleles and (2) the intensity of phenotypic resistance of the vectors. In anticipation of a national scale-up of PBO-ITN deployment by Benin’s NMCP, we evaluated resistance intensity levels with and without pre-exposure to PBO in malaria vectors from areas with diverse ecologies thought to have high levels of insecticide resistance.

## Methods

### Study sites

**Fig. 1:**
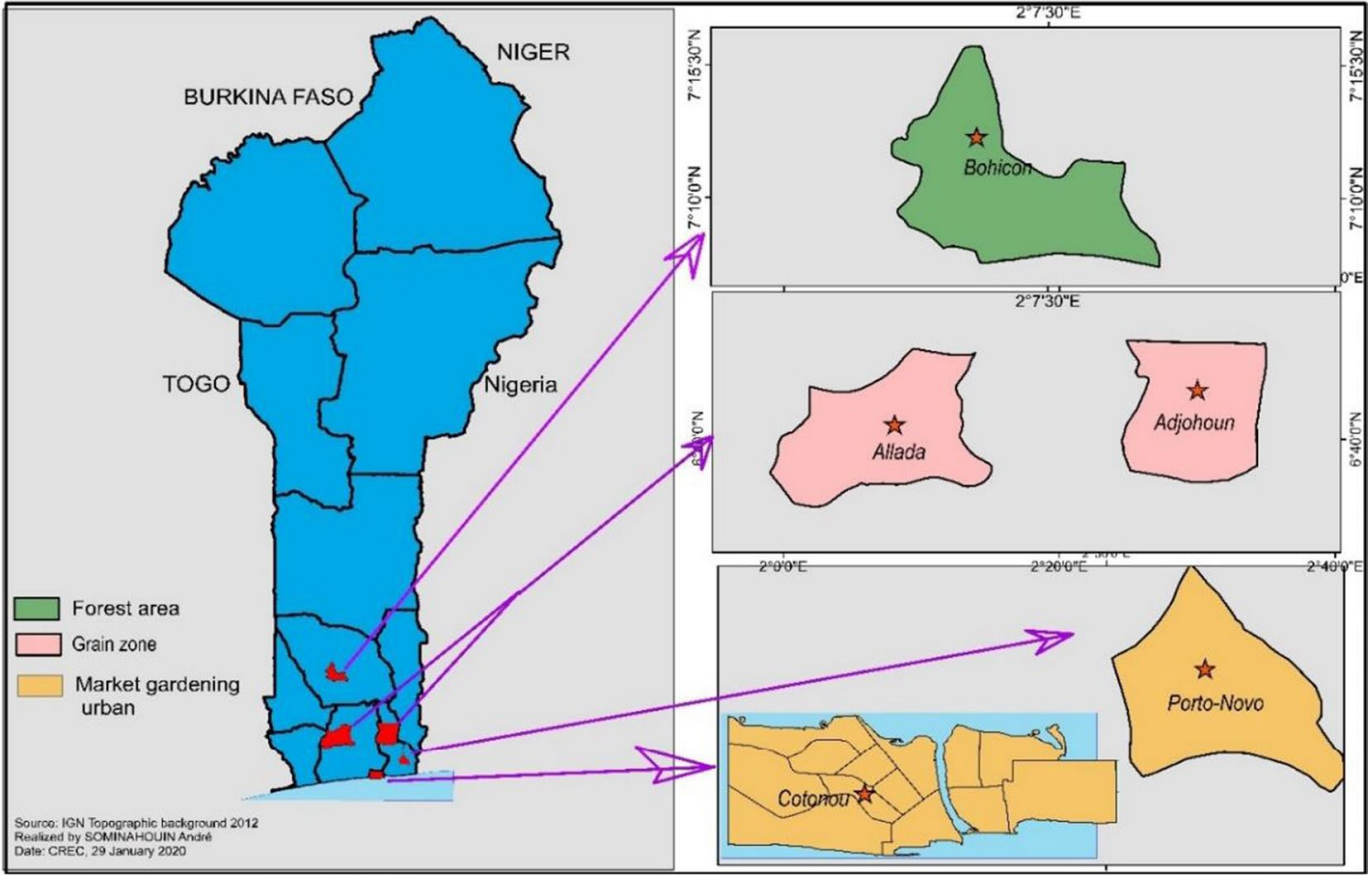
Study sites

This study was carried out from August 2017 to July 2018 in five communes in southern Benin: Cotonou, Porto-Novo, Allada, Adjohoun, and Bohicon (Fig. 1). These communes were selected based on their suspected insecticide resistance status and diverse ecology. Cotonou and Porto-Novo are urban areas located respectively in the Littoral and Ouémé departments in which IRS has never been implemented but where the population uses impregnated mosquito nets (LLINs), aerosol cans, and smoke coils to protect themselves against mosquito bites. In these communes, market gardening areas are well developed and represent the prolific larval habitats of *An. gambiae* s.l. The communes of Allada, Bohicon, and Adjohoun, located respectively in the departments of Atlantic, Zou, and Ouémé, are characterized by the production of food and cereal crops. Maize and tubers are the most widely cultivated crops and represent the staple foods of the populations in these communes. In addition to cereals and tubers, the commune of Adjohoun cultivates vegetables in large quantities during the dry season, as its soil is enriched by the silt deposited by annual floods caused by seasonal rainfall peaks in June, which is very favorable for vegetable and out-of-season crops. The commune of Bohicon is a forest area. The yearly average temperature in the south of Benin ranges from 21.9 to 32.8 °C and rainfall from 900 to 1100 mm [26].

### Larval collections

Larval collections were carried out with a dipper, trays, bowls, and a very fine mesh sieve (repurposed unused coffee filter) in the five communes. Larvae were brought to the insectary of Centre de Recherche Entomologique de Cotonou (CREC). Established procedures were used to optimize growth and prevent cannibalism [27]. The larvae were fed ground cat food. Each tray was covered with a veil and kept in the larval room of the insectary. The photoperiod was ensured by fluorescent lamps illuminating the larval room from 6:00 p.m. to 6:00 a.m. Pupae were collected and inserted into 30 × 30 × 30 cm mosquito cages to maximize the collection of emerged adults. Mosquitoes were fed a 10% honey solution before susceptibility testing. Adult mosquitoes were identified with a magnifying glass and an *Anopheles* species identification key [28, 29].

### WHO insecticide resistance intensity and synergist assay

For all WHO susceptibility assays, approximately 100 to 150 adult female *An. gambiae* s.l., aged 2 to 5 days, reared from larvae from each of the study sites were used in assays. For intensity assays, adult *An. gambiae* s.l. females from each of the communes were exposed to the diagnostic dose (1×), and 2×, 5×, and 10× the diagnostic dose of permethrin and deltamethrin to determine the level of resistance in the mosquito population. For synergist assays, mosquitoes were pre-exposed to papers treated with 4% PBO and then exposed to different doses of permethrin and deltamethrin to assess the level of oxidase involvement in the resistance of these vectors to pyrethroids. Each assay included control mosquitoes exposed to the paper impregnated only with silicone oil. The WHO procedure was followed for both intensity and synergist assay [30]. Mosquito mortality was observed and recorded after 24 h. Dead and live mosquitoes from the tests were stored in Eppendorf tubes with silica gel for molecular testing. All impregnated papers were obtained from WHO at the standard doses. Although the 2× deltamethrin (0.1%) and 2× permethrin (1.5%) are not part of WHO’s routine insecticide concentration offerings, WHO did provide this concentration of these two pyrethroids by special request.

### Molecular analysis of mosquito species and insecticide resistance of *An. gambiae* s.l.

Molecular analyses were carried out on 100 mosquitoes per commune to identify the different mosquito species and evaluate the genes involved in *An. gambiae* s.l. resistance to insecticides. Using established protocols, dead and live mosquitoes from the WHO insecticide susceptibility tests were analyzed for the identification of sibling species of the *An. gambiae* complex [31] and the frequency of the resistance alleles, *kdr-west* [12] and *ace-1*R [32].

### Biochemical assays of detoxifying enzymes in each *An. gambiae* s.l. population

To identify the different detoxification enzymes including the esterases, oxidases, and GSTs involved in vector resistance to insecticides, biochemical assays were carried out on 50 whole-bodied unexposed (mosquitoes from bioassay controls) females (2–5 days old) from the same mosquito population as that used for the WHO susceptibility test. Protocols described in the MR4 manual [33] were followed. Total soluble protein analysis was done to correct for any variation in enzyme levels based on size differences between the different mosquito populations. Results from these biochemical tests were compared with a laboratory Kisumu susceptible strain of the same age (2–5 days old).

### Statistical analysis

The resistance status of malaria vectors was determined according to WHO criteria [30] as follows:

-  ≥ 98% mortality 24 h after insecticide exposure, the *An. gambiae* s.l. population is susceptible
- Between 90 and 97% mortality 24 h after insecticide exposure, the *An. gambiae* s.l. population is suspected of being resistant (requires confirmation)
-  < 90% mortality 24 h after insecticide exposure, the *An. gambiae* s.l. population is resistant

No mortality was recorded in controls. Therefore, Abbott's formula was not necessary to correct the mortality rates.

To determine the increased mortality caused by PBO pre-exposure followed by deltamethrin or permethrin exposure, the mortality rate difference and 95% confidence interval were calculated for deltamethrin and permethrin groups exposed and not exposed to PBO at all study sites. Pearson’s *χ*^2^ test was done to determine the level of significance in the insecticide groups at all study sites. The allele frequencies of *kdr* L1014F and *Ace-1*R G119S were calculated to assess their variability in the populations, as follows:

$$ F \, \left( R \right) \, = \, \left[ {2n{\text{RR }} + \, n{\text{RS}}} \right] \, / \, \left[ {2\left( {n{\text{RR }} + \, n{\text{RS }} + \, n{\text{SS}}} \right)} \right] $$

where F(R) is the frequency of resistance, n is the number of mosquitoes of a given genotype, RR is the homozygous resistant genotype, RS is the heterozygous resistant genotype, and SS is the susceptible genotype [12, 32].

Analysis of variance (ANOVA) was used to assess the variation in enzyme activity in each locality. Tukey's multiple comparison test was used to compare enzyme activity between susceptible Kisumu laboratory mosquitoes and those collected in the field. All statistical analyses were performed using R 3.3.2 software.

## Results

### Deltamethrin and permethrin resistance intensity in *An. gambiae* s.l.

**Fig. 2:**
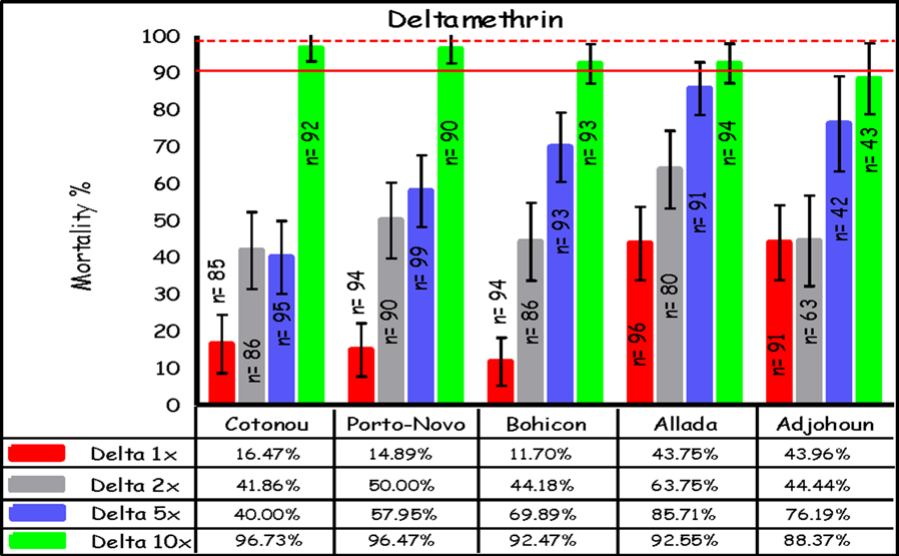
Mortality of *An. gambiae* s.l. after exposure to increasing doses of deltamethrin, using the WHO tube test (error bars represent the standard errors; the broken red line is the threshold for suspected resistance, the solid line indicates the threshold for confirmed resistance)

A total of 1697 female *An. gambiae* s.l. were exposed to different deltamethrin doses in all study sites. At 24 h post-exposure, the mortality rate at the diagnostic dose 1× varied across sites. The lowest mosquito mortality rates at 1× were observed in Bohicon (12%), Porto-Novo (15%), and Cotonou (16%). Mortality rates in Adjohoun (44%) and Allada (44%) were higher but were well below the WHO threshold of ≥ 98% mortality for susceptible mosquito populations. Though mortality increased at all sites with increasing dosage of deltamethrin, the WHO threshold of ≥ 98% mortality was still not achieved, even at 10× the diagnostic dose; this suggests high-intensity resistance to deltamethrin (Fig. 2).

**Fig. 3:**
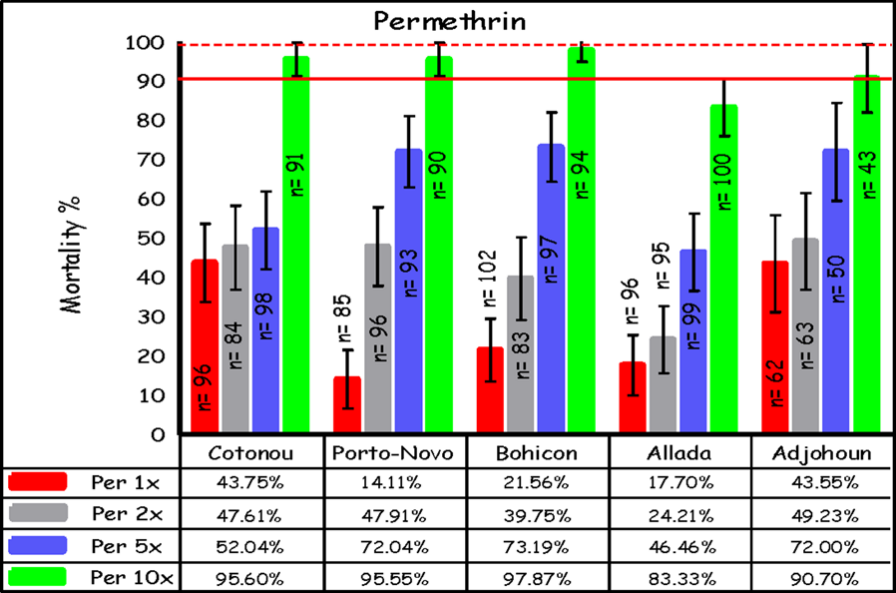
Mortality of *An. gambiae* s.l. after exposure to increasing doses of permethrin, using the WHO tube test (error bars represent the standard errors; the broken red line is the threshold for suspected resistance, the solid line indicates the threshold for confirmed resistance)

Results with permethrin were similar to those observed with deltamethrin. A total of 1296 female *An. gambiae* s.l. were exposed to the different doses in all study sites. The lowest mortality rates at the diagnostic dose (1×) were observed in Porto-Novo (14%), Allada (18%), and Bohicon (22%). The mortality rate in both Adjohoun and Cotonou was 44%. Likewise, at all sites, permethrin susceptibility increased as mosquitoes were exposed to higher doses of insecticide; however, none of the assays achieved the WHO threshold of ≥ 98% mortality, suggesting a high intensity of permethrin resistance (Fig. 3).

### Susceptibility of *An. gambiae* s.l. to deltamethrin and permethrin after PBO pre-exposure

**Fig. 4:**
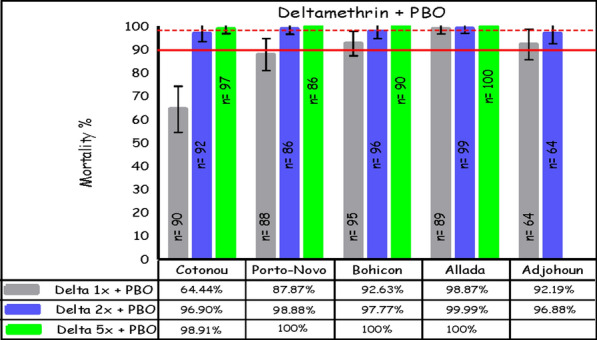
The mortality rate for *An. gambiae* s.l. after exposure to increasing doses of deltamethrin combined with the synergist PBO, using the WHO tube test (error bars represent the standard errors; the broken red line is the threshold for suspected resistance, the solid line indicates the threshold for confirmed resistance)

**Fig. 5:**
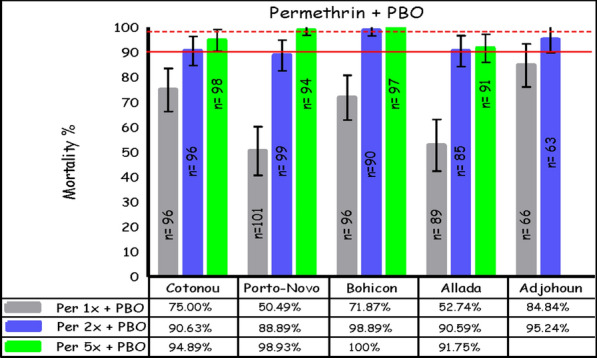
The mortality rate for *An. gambiae* s.l. after exposure to increasing doses of permethrin associated with the synergist PBO, using the WHO tube test (error bars represent the standard errors of the mortality rate at the y-axis; below the broken red line suggests suspected resistance, below the solid line signifies confirmed resistance)

After PBO pre-exposure, mortality after exposure to deltamethrin ranged from 65% in Cotonou to 99% in Allada at 1×; from 97% in Cotonou and Adjohoun to 100% in Allada at 2×; and from 99% in Cotonou to 100% in Bohicon, Allada, and Porto-Novo at 5× (Fig. 4). For permethrin, pre-exposure to PBO also led to an increase in *An. gambiae* s.l. mortality, with rates ranging from 50% in Porto-Novo to 85% in Adjohoun at 1×; from 89% in Porto-Novo to 99% in Bohicon at 2×; and from 92% in Allada to 100% in Bohicon at 5× (Fig. 5).

For deltamethrin, the mortality rate difference ranged from 48% in Cotonou to 81% in Bohicon; across sites, there was a significant increase of 61% (*p* < 0.001) in the mortality rate for *An. gambiae* s.l. after pre-exposing mosquitoes to PBO and then exposing them to deltamethrin at the diagnostic dose (Table 1). For permethrin, the mortality rate difference ranged from 31% in Cotonou to 50% in Bohicon; across sites, there was a significant increase of 39% (*p* < 0.001) in the mortality rate in *An. gambiae* s.l. after pre-exposing mosquitoes to PBO and then exposing them to permethrin at the diagnostic dose (Table 2).

**Table 1:** Mortality rate difference and 95% confidence interval (CI) for 1× deltamethrin with and without pre-exposure to PBO

Site	Insecticide	Dead	Alive	Total	Mortality rate (%)	Rate difference*	95% CI
Adjohoun	PBO + 1× Deltamethrin	59	05	64	92	48	36–60
	1× Deltamethrin	40	51	91	44		
Allada	PBO + 1× Deltamethrin	88	01	89	99	55	45–65
	1× Deltamethrin	42	54	96	44		
Bohicon	PBO + 1× Deltamethrin	88	07	95	93	81	73–89
	1× Deltamethrin	11	83	94	12		
Cotonou	PBO + 1× Deltamethrin	58	32	90	64	48	35–61
	1× Deltamethrin	14	71	85	16		
Porto-Novo	PBO + 1× Deltamethrin	77	11	88	88	73	63–81
	1× Deltamethrin	14	80	94	15		
All sites	PBO + 1× Deltamethrin	370	56	426	87	61	56–66
	1× Deltamethrin	121	339	460	26		

*Differences in mortality rates between 1× deltamethrin and PBO + 1× deltamethrin were significant (*p* < 0.001) at all sites using the *χ*^2^ test

**Table 2:** Mortality rate difference and 95% confidence interval (CI) for 1× permethrin with and without pre-exposure to PBO

Site	Insecticide	Dead	Alive	Total	Mortality rate (%)	Rate difference	95% CI
Adjohoun	PBO + 1× Permethrin	56	10	66	85	41	26–56
	Permethrin	27	35	62	44		
Allada	PBO + 1× Permethrin	47	42	89	53	35	22–48
	Permethrin	17	79	96	18		
Bohicon	PBO + 1× Permethrin	69	27	96	72	50	38–62
	Permethrin	22	80	102	22		
Cotonou	PBO + 1× Permethrin	72	24	96	75	31	18–44
	Permethrin	42	54	96	44		
Porto-Novo	PBO + 1× Permethrin	51	50	101	50	36	24–49
	Permethrin	12	73	85	14		
All sites	PBO + 1× Permethrin	295	153	448	66	39	26–52
	Permethrin	120	321	441	27		

*Differences in mortality rates between 1× permethrin and PBO + 1× permethrin were significant (*p* < 0.001) at all sites using the *χ*^2^ test

### Species identification and allelic frequency of *kdr-west* and *ace-1*R genes

Species identification results revealed two species of *An. gambiae* s.l. in Porto-Novo, Bohicon, Cotonou, and Adjohoun, with a predominance of *An. coluzzii* in Porto-Novo (79%) and Adjohoun (96%), and *An. gambiae* s.s. in Cotonou (98%) and Bohicon (56%). In Allada, *An. coluzzii* was the only species (100%) of the *An. gambiae* complex recorded. The polymerase chain reaction (PCR) results also showed a high frequency of the *Kdr-west* mutation in the five study sites; the allelic frequency for *An. gambiae* s.s. in Porto-Novo, Bohicon, Adjohoun, and Cotonou was 0.76, 0.82, 1.00, and 0.92, respectively. The same result was observed in *An. coluzzii*, with 0.75 in Cotonou, 0.82 in Porto-Novo, 0.84 in Bohicon, 0.87 in Adjohoun, and 0.76 in Allada. However, no significant difference in allelic frequency was recorded between *An. gambiae* and *An. coluzzii* from different sites (Table 3). The allelic frequency of *Ace-1*R was very low in both species across the five study sites, with values ranging from 0.001 in Bohicon to 0.03 in Cotonou for *An. gambiae*, and from 0.001 in Bohicon to 0.01 in Adjohoun and Allada for *An. coluzzii* (Table 3).

**Table 3:** Allelic frequencies of *Kdr* and *Ace-*1 in *Anopheles gambiae* s.l. of Cotonou, Porto-Novo, Bohicon, Allada, and Adjohoun

Locality	Species	No. tested	Mutation *Kdr west*					Mutation *Ace-1*R				
			RR	RS	SS	Freq^†^	p^*^	RR	RS	SS	Freq^†^	p^*^
Cotonou	*An. coluzzii*	2	1	1	0	0.750^a^	0.738	0	0	2	0.000^a^	1
	*An. gambiae*	98	86	9	3	0.920^a^		0	6	92	0.030^a^	
Porto-Novo	*An. coluzzii*	79	54	21	4	0.820^a^	0.566	0	1	78	0.006^a^	1
	*An. gambiae*	21	13	6	2	0.760^a^		0	0	21	0.000^a^	
Bohicon	*An. coluzzii*	44	33	8	3	0.840^a^	0.862	0	1	43	0.001^a^	0.791
	*An. gambiae*	56	41	10	5	0.820^a^		0	3	53	0.002^a^	
Adjohoun	*An. coluzzii*	96	76	16	4	0.870^a^	0.609	0	2	94	0.010^a^	1
	*An. gambiae*	4	4	0	0	1.000^a^		0	0	4	0.000^a^	
Allada	*An. coluzzii*	100	66	20	14	0.760^a^	1	0	2	98	0.010^a^	1
All sites	*An. coluzzii*	321	230	66	25	0.820	NA	0	6	315	0.010	NA
	*An. gambiae*	179	144	25	10	0.870		0	9	170	0.030	

*No.* number, *An* *Anopheles*, *Freq* frequency, *NA* not applicable

*p-value: based on *χ*^2^ test to compare the frequencies of both species by study site

^†^For the same species, the study site with frequencies bearing the same letter do not differ significantly (*p* > 0.05)

### Enzymatic activity in *An. gambiae* s.l. from each commune

**Fig. 6:**
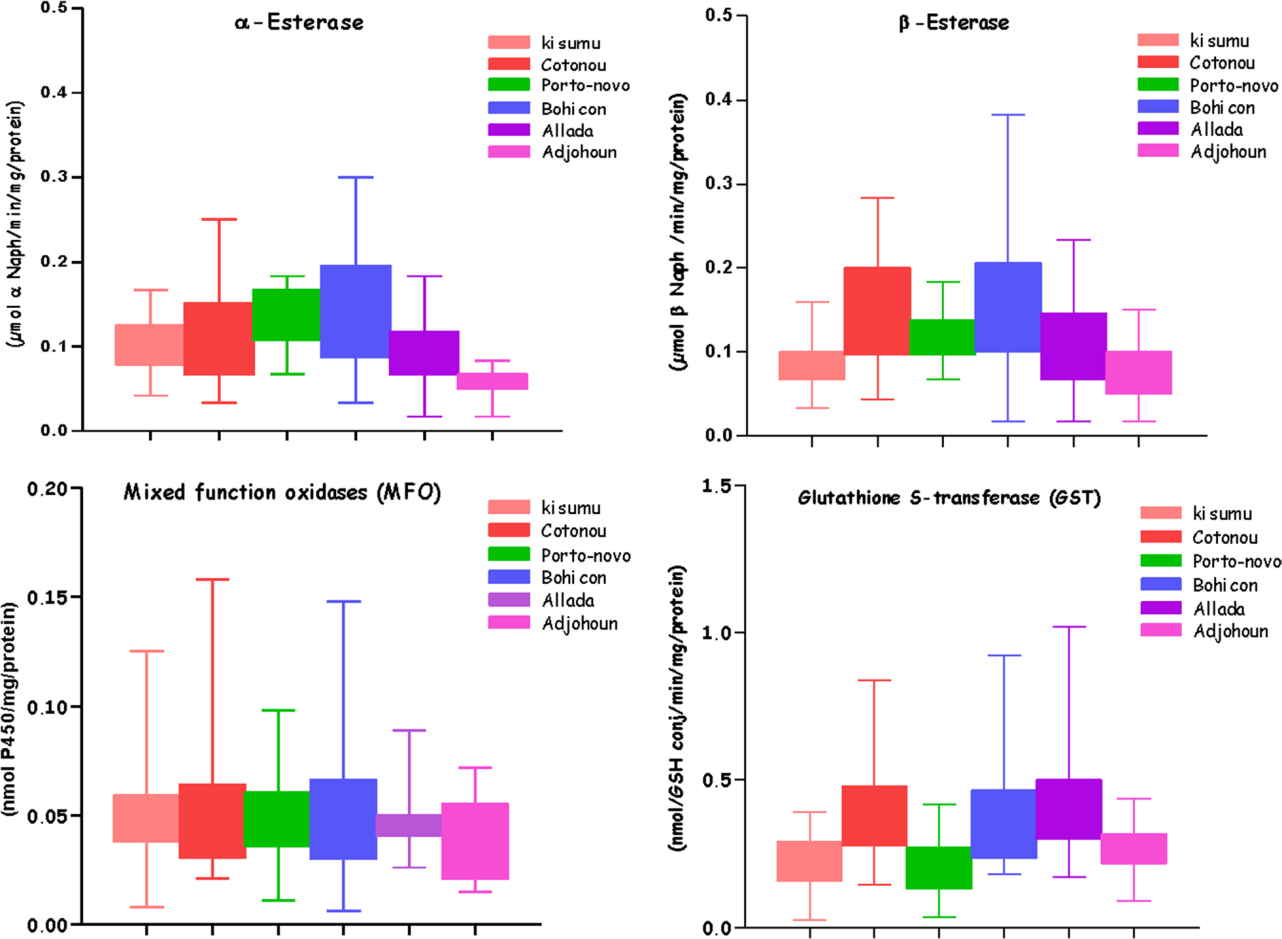
Box-and-whisker plot of the detoxification enzymes [nonspecific esterases (α- and β-esterases), mixed-function oxidases (MFO), and glutathione S-transferase (GST)] in the laboratory colony strain, *An. gambiae* s.s. (Kisumu) and wild *An. gambiae* s.l. populations from Adjohoun, Allada, Bohicon, Cotonou, and Porto-Novo. (The boxes represent the 25th and 75th percentiles of individual mosquito measurements; the horizontal black line within the box indicates the median, and the x indicates the mean)

The analysis of the enzymatic activity of *An. gambiae* s.l. compared to the Kisumu susceptible reference strain showed overexpression of nonspecific α- and β-esterases in Cotonou (*p*_α_ = 0.5607, *p*_β_ = 0.0001), Porto-Novo (*p*_α_ = 0.0001, *p*_β_ < 0.0001), and Bohicon (*p*_α_ = 0.0009, *p*_β_ < 0.0001). In the commune of Allada, the expression of these enzymes was similar (*p*_α_ = 0.1519, *p*_β_ = 0.1803), unlike in Adjohoun (*p*_α_ < 0.0001, *p*_β_ = 0.0307), where under-expression of these enzymes was observed compared to the Kisumu susceptible strain. For oxidases, expression similar to that of the Kisumu susceptible strain (*p* > 0.5493) was recorded in all study sites. For GST, enzymatic activity was significantly higher in field strains compared to the Kisumu strain in all study sites (*p* < 0.0001) except Porto-Novo (*p* = 0.4613) and Adjohoun (*p* = 0.1612), where no significant differences were observed (Fig. 6; Table 4).

**Table 4:** Result of statistical analysis of mean (± SE) mixed-function oxidase, glutathione S-transferase, and esterase activity in *An. gambiae* s.l. populations

Strain	α-Esterase	β-Esterase	MFO	GST
	(µmol α-Naph/min/mg protein)	(µmol β-Naph/min/mg protein)	(nmol/P450/min/mg protein)	(nmol/GSH conj/min/mg protein)
Kisumu	0.101 ± 0.010^a^	0.091 ± 0.009^a^	0.052 ± 0.008^a^	0.222 ± 0.027^a^
Cotonou	0.113 ± 0.021^a^	0.151 ± 0.024^b^	0.052 ± 0.011^a^	0.398 ± 0.062^b^
Porto-Novo	0.132 ± 0.010^b^	0.122 ± 0.009^a^	0.050 ± 0.006^a^	0.209 ± 0.034^a^
Bohicon	0.148 ± 0.020^b^	0.164 ± 0.023^b^	0.053 ± 0.009^a^	0.365 ± 0.051^b^
Allada	0.089 ± 0.011^a^	0.106 ± 0.015^a^	0.048 ± 0.004^a^	0.418 ± 0.052^b^
Adjohoun	0.058 ± 0.009^c^	0.072 ± 0.016^a^	0.039 ± 0.008^a^	0.261 ± 0.044^a^
One-way ANOVA	*F* = 14.42; *df* = 5, 221;	*F* = 13.88; *df* = 5, 221;	*F* = 01.05; *df* = 5, 221;	*F* = 16.34; *df* = 5, 221;
	*p* < 0.0001	*p* < 0.0001	*p* < 0.5728	*p* < 0.0001

*MFO*  mixed-function oxidases, *GST* glutathione S-transferase, *GSH* glutathione. Means followed by different letters are significantly different, *p* < 0.05, Tukey’s test

*Significant increase in mean differences compared to the laboratory reference strain, *p* < 0.05, *t* test. In the same column, the values indexed to the same letter are not significantly different

## Discussion

This study showed a high intensity of deltamethrin and permethrin resistance in *An. gambiae* s.l. using different insecticide doses in southern Benin. It also showed an increase in mortality from deltamethrin and permethrin after pre-exposure to PBO. Molecular analysis showed high levels of *kdr* resistance alleles in mosquito populations but low levels of *ace-1* alleles. High biochemical test levels showed overexpression of GSTs and esterases but not oxidases.

Before 2020, all ITNs distributed in Benin’s mass campaigns or through continuous distribution channels consisted only of standard ITNs. In this study, the resistance of *An. gambiae* s.l. to the diagnostic dose (1×) of permethrin and deltamethrin was observed in all southern Benin tested sites, confirming the resistance status of *An. gambiae* s.l. reported in past investigations in Benin [2–4, 7, 34]. In most sites, *An. gambiae* s.l. clearly showed a high tolerance to 2×, 5×, and 10× the diagnostic dose of permethrin and deltamethrin, suggesting high resistance as defined by WHO [30]. The results of this study are alarming, because insecticide-based vector control failure is thought to occur when mosquitoes exhibit insecticide resistance at 5× or 10× of the diagnostic dose [30]. Though ITNs may still act as a physical barrier to infectious mosquito bites, the killing effect of the net may be severely diminished. National coverage of LLINs is a major part of the malaria control strategy in Benin’s National Malaria Control Program (NMCP). These findings indicate that the effectiveness of the sole vector control strategy—mass distribution of mosquito bed nets treated with pyrethroids only—in southern Benin could be threatened by high pyrethroid resistance in the mosquito population [35]. In response to this reduced effectiveness of insecticides, NMCP distributed PBO-ITNs in 2020 in about 19 communes (25%) selected from southern to central Benin to mitigate the potential negative impact of resistance on insecticide effectiveness. In line with the vision of increasing the effectiveness of vector control tools, Benin plans to cover more than 60% of the country's 77 communes in 2023 with PBO-ITNs in the next mass distribution campaign.

Similar to other studies in Benin [3, 9, 24], there was a significant increase in mortality from deltamethrin and permethrin at the diagnostic dose after PBO pre-exposure; additionally, the increase in mortality was greater for PBO plus deltamethrin than PBO plus permethrin at the diagnostic dose. However, in this study, complete restoration to the WHO threshold of susceptibility (≥ 98% mortality) at the diagnostic dose [30] occurred only for PBO plus deltamethrin in Allada. In some sites, even exposure to 5× permethrin + PBO was not able to completely restore insecticide susceptibility to WHO threshold levels. Nonetheless, there was still a significant increase in mortality in the mosquitoes pre-exposed to PBO versus mosquitoes exposed to deltamethrin or permethrin only.

In addition to the high prevalence of permethrin and deltamethrin insecticide resistance in *An. gambiae* s.l., the *kdr-west* allele was found at high frequencies, ranging from 75 to 100%. This suggests near fixation of the *kdr-west* resistance allele in the southern Benin vector population. Previous studies on the spatial dynamics of insecticide resistance in Benin [7, 10, 11, 36] have characterized the distribution of *kdr-west* in time and space. These studies have suggested that various ecological pressures shape the mosquito populations. Though this was not investigated in the present study, the high level of resistance of *An. gambiae* s.l. in southern Benin could be explained by the high selective pressure from different uses of insecticides, such as through (1) protective measures taken against adult mosquito bites (i.e. LLINs or spray aerosols), (2) agricultural insecticide runoff into mosquito larval sites, or (3) the combined effect of the two. It is likely that sustained ecological pressure continues to expand the *kdr-west* alleles in the mosquito population and potentially limits the effectiveness of vector control strategies such as LLINs [37].

The effect of PBO combined with pyrethroids implies that cytochrome P450 mono-oxygenases were highly involved in the resistance of *An. gambiae* s.l. to deltamethrin and permethrin in all the study sites [38, 39]. However, the results of the enzymatic assays, which were performed on the same vector population and compared to the *An. gambiae* s.s. Kisumu (insecticide-susceptible strain), did not show an over-expression of the oxidases in the different study sites. It has been speculated that the interaction between fitness cost and resistance conferred by mutations in the oxidase genes may “facilitate the loss of overproduction” [40, 41], since studies have shown that overproduction of P450 oxidases can affect “endogenous molecules in insects” [42]. This may explain why PBO pre-exposure increased mortality but was not over-expressed in biochemical assays. It is not clear whether there is another mechanism of resistance not elucidated in this study. It may be useful to conduct follow-up tests to explain this anomaly. There was high activity of GSTs, which suggests that in wild populations of *An. gambiae* s.l. in Cotonou, Bohicon, and Allada, these enzymes could play a major role in the resistance to pyrethroids due to the detoxification of products derived from peroxidation of lipids induced by pyrethroids [43]. Therefore, these enzymes (GST) could increase phenotypic resistance by widening the spectrum of insecticide resistance mechanisms [44]. These results reinforce the idea that there could be other insecticide resistance mechanisms in *An. gambiae* s.l. in addition to *kdr*, the oxidases, and GSTs, which are involved in the resistance of this vector to pyrethroids.

## Conclusion

This study identified *An. gambiae* s.l. with high resistance levels to pyrethroids in southern Benin. The level of resistance in *An. gambiae* s.l. is alarming with respect to the implications for pyrethroid-based control strategies. While standard LLINs provide a physical barrier to mosquito bites, the killing effect of the net may be limited by the resistance levels in southern Benin communes. The increase in the mortality rates when mosquitoes were exposed to deltamethrin and permethrin after exposure to PBO suggests that the use of PBO-ITNs (PermaNet 3.0 and Olyset Plus) may mitigate the effect of insecticide resistance and maintain the gains achieved with the mass distribution of standard LLINs.

## Data Availability

The data used and/or analyzed in this study are available from the corresponding author on reasonable request.
